# Permanent Bilateral Carotid Filters for Stroke Prevention in Atrial Fibrillation

**DOI:** 10.1007/s11886-020-01388-w

**Published:** 2020-09-10

**Authors:** Tom De Potter, Ofer Yodfat, Guy Shinar, Avraham Neta, Vivek Y. Reddy, Petr Neuzil, Roland Veltkamp, Stuart J. Connolly

**Affiliations:** 1 Cardiovascular Center, OLV Hospital, Moorselbaan 164, B-9300 Aalst, Belgium; 2 Javelin Medical Ltd., Yokne’am, Israel; 3 Helmsley Electrophysiology Center, Division of Cardiology, Icahn School of Medicine at Mount Sinai, New York, NY USA; 4 Department of Cardiology, Homolka Hospital, Prague, Czech Republic; 5 Department of Brain Science, Imperial College London, London, UK; 6 Department of Neurology, Alfried Krupp Krankenhaus, Essen, Germany; 7 Department of Neurology, University Heidelberg, Heidelberg, Germany; 8 Population Health Research Institute, McMaster University, Hamilton, Ontario Canada

**Keywords:** Atrial fibrillation, Carotid filter, Common carotid artery, Embolic protection, Stroke prevention

## Abstract

**Purpose of Review:**

A novel permanent carotid filter device for percutaneous implantation was developed for the purpose of stroke prevention. In this review, we cover rationale, existing preclinical and clinical data, and potential future directions for research using such a device.

**Recent Findings:**

The Vine™ filter was assessed for safety in sheep and in 2 observational human studies, the completed CAPTURE 1 (*n* = 25) and the ongoing CAPTURE 2 (planned *n* = 100). CAPTURE 1 has shown high procedural and long-term implant safety. A control group was not available for comparison.

**Summary:**

A mechanical filter for permanent stroke prevention can be implanted bilaterally in the common carotid artery safely and efficiently. A randomized trial is planned for 2021 (*n* = 3500, INTERCEPT) to demonstrate superiority of a filter + anticoagulation strategy over anticoagulation alone in patients at high risk for ischemic stroke.

## Introduction

Stroke prevention in atrial fibrillation (AF) patients is an important medical need with major consequences if left unmet. In the USA, AF prevalence is > 6 million, with an incidence of 1.2 million cases per year [1, 2]. Nearly 800,000 Americans suffer from stroke each year, and one out of three is associated with AF (67% of them with prior AF) [3].

Stroke risk in AF patients is commonly estimated using the CHA_2_DS_2_-VASc scoring system [4]. In AF patients not taking oral anticoagulants (OAC), the average annual stroke risk is ~ 5%/year (2% and 11% in CHA_2_DS_2_-VASc = 2 and 9, respectively) [5–8]. Warfarin and new oral anticoagulants (NOAC) reduce the stroke risk in AF patients by at least 65% [9]. However, in some high-risk groups of AF patients, the annual ischemic stroke risks remain high even when receiving OAC, with estimates of 8.9% (first year after recent stroke while on OAC) [10•], 5.6% (after recent stroke and age > 75) [11], 4.4% (within 90 days of stroke) [12], 2.3–3.2% (with any remote history of stroke (> 90 days)) [13–15], and 1.8% (age > 75) [16].

One of the reasons that stroke risk can remain high in some AF patients is that long-term adherence to OAC treatment is sub-optimal. The rate of temporary OAC discontinuation over 3 days was 63% in the ENGAGE AF-TIMI 48 randomized controlled trial (edoxaban vs. warfarin) [17]. In a retrospective cohort analysis of a large US commercial insurance database, fewer than half (47.5%) of the patients had ≥ 80% days covered by NOAC [18]. The resulting stroke risk during interruption and discontinuation was substantially increased. In the ROCKET AF trial (rivaroxaban vs. warfarin), stroke risks during rivaroxaban temporary interruptions (3–30 days) and permanent discontinuations (> 30 days) were 6.2%/year and 25.6%/year, respectively [19].

There is substantial necropsy evidence demonstrating that total anterior stroke only occurs if the proximal stem (M1) of the middle cerebral artery (MCA) is occluded [20]. Partial anterior stroke consists of more restricted cortical infarcts due to occlusion of the upper or lower divisions (M2) of the MCA [21]. The underlying cause of these strokes, in most cases, is a proximally originating embolus rather than in situ thrombosis. Thus, in AF patients, anterior circulation strokes (~ 80% of strokes) are likely attributable to emboli originating from central circulatory system. Anterior circulation strokes are more severe than lacunar strokes and posterior circulation strokes [22]. Compared with lacunar stroke, the odds ratios of death for total anterior circulation stroke, partial anterior circulation stroke, and posterior circulation stroke are 5.73, 1.65, and 2.22, respectively, and the odds ratios of disability are 3.27, 1.73, and 0.88, respectively. Thus, protection against anterior circulation strokes in AF patients is a major priority, compared with strokes in other distributions.

The calibers (diameters) of the middle cerebral and anterior cerebral arteries and their first branches are > 1.5 mm [23–28]. Thus, a permanent, bilateral, common carotid artery (CCA) filter that can exclude emboli of > 1.5 mm in size could therefore potentially reduce the risk of most major anterior circulation embolic strokes.

**Fig. 1:**
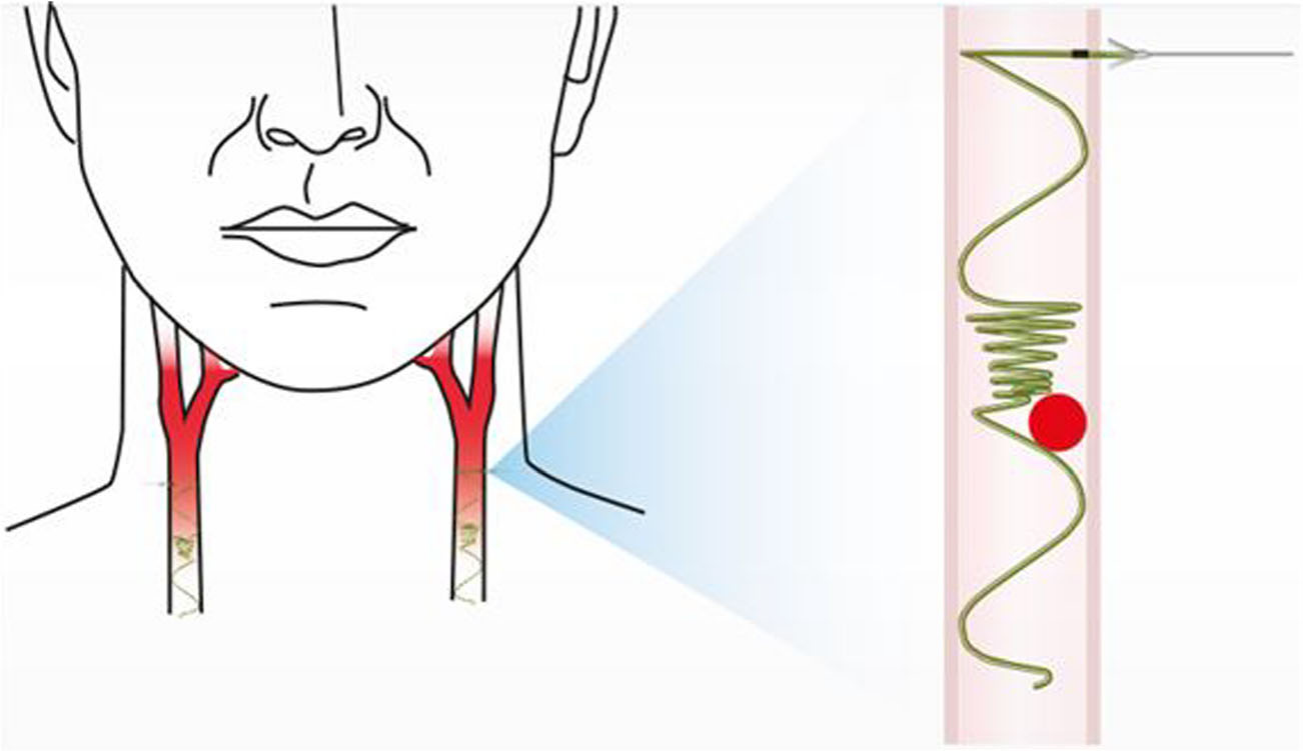
Typical implant position in the common carotid artery. Illustration shows potential captured thrombus location (red circle)

Accordingly, a CCA filter device has been designed to be a permanent implant inserted into the common carotid artery to capture emboli > 1.5 mm in size and thus prevent their propagation to the brain (Fig. 1). In AF patients, it is designed to prevent emboli from reaching the anterior cerebral circulation and thus to prevent most anterior circulation strokes (representing about 80% of stroke in AF patients). The implant is being developed as a means of providing permanent stroke prevention in AF patients at high stroke risk, despite ongoing treatment with OAC.

## Device Description

**Fig. 2:**
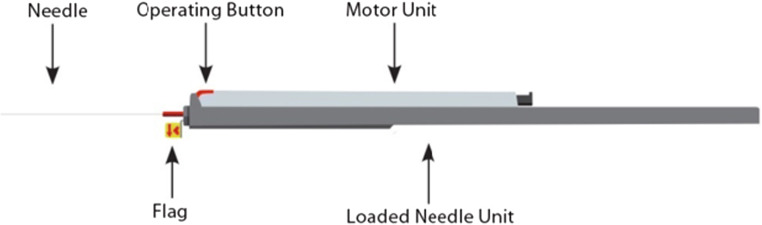
The Vine™ Embolic Protection System

The Vine™ Embolic Protection System (“system,” Fig. 2) consists of the Vine™ CCA Filter (“implant”) and a Vine™ Inserter (inserter).

**Fig. 3:**
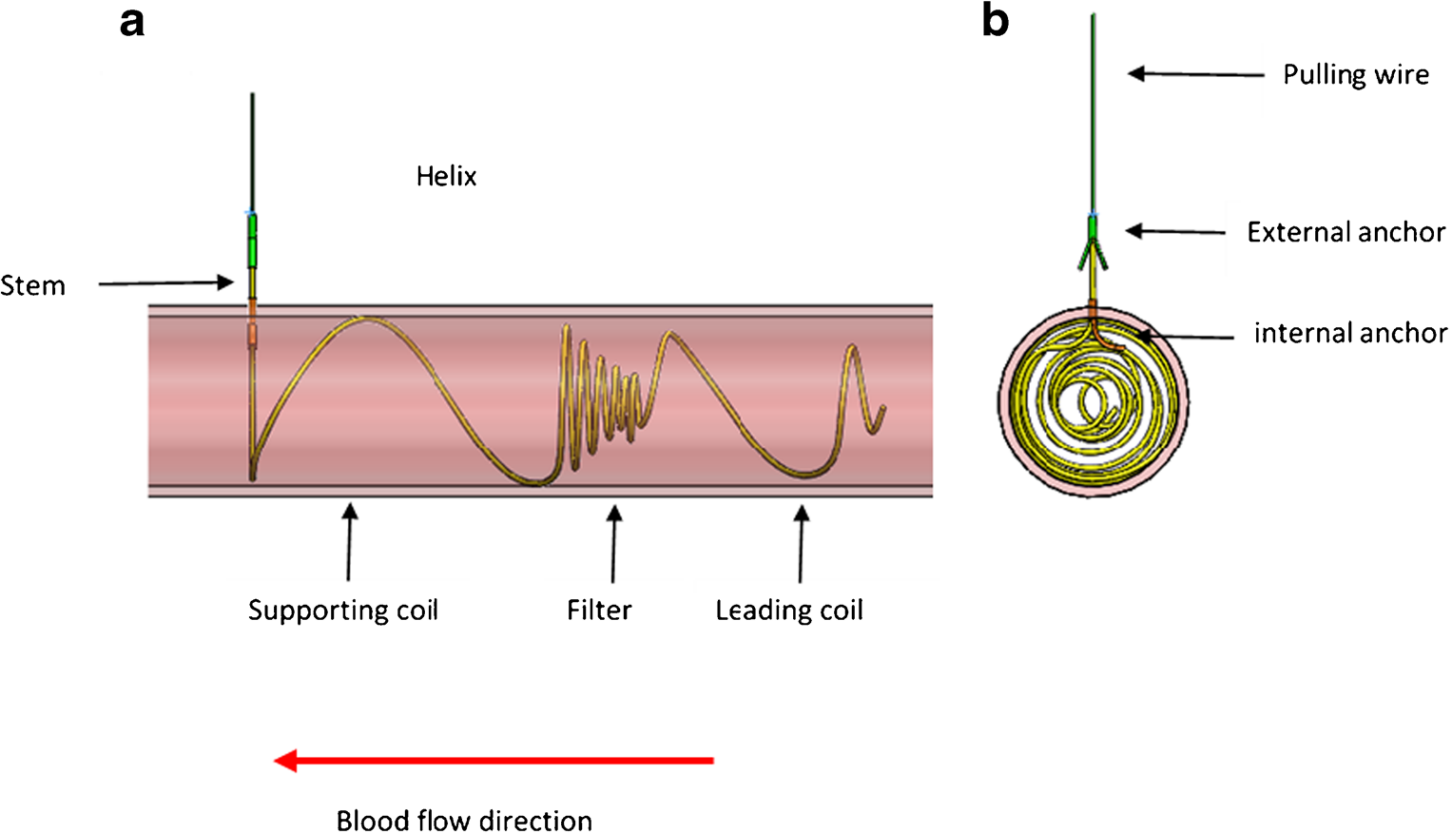
The implant: longitudinal (**a**) and cross section (**b**) views

The implant is made of a super-elastic single nitinol wire—a monofilament structure capable of assuming a substantially linear shape in the un-deployed state, and a functional shape, once deployed. In the deployed state, the implant (Fig. 3) comprises a helix that resides within the CCA lumen and a linear stem that traverses the CCA wall. The helix includes 3 segments: supporting coils, filtering portion (“filter”), and leading coils. The filtering portion has a cone shape facing upstream (toward the heart) and includes coils that are spaced approximately 0.85 mm apart. During deployment, the implant is fixed in place by anchors (internal and external). The implant is provided in 10 sizes (5.5–10 mm, at 0.5-mm intervals) to accommodate CCA diameters of 4.8–9.8 mm. In terms of anatomy, extreme tortuosity (at the discretion of the implanting physician) or reverse tapering (an increasing diameter cranially) would present a contra-indication to implantation. The implant may be retrieved without surgery using a pulling wire that is connected to the stem. At up to 4 hours, the pulling wire is cut at the skin level after which surgery would be required for removal.

**Fig. 4:**
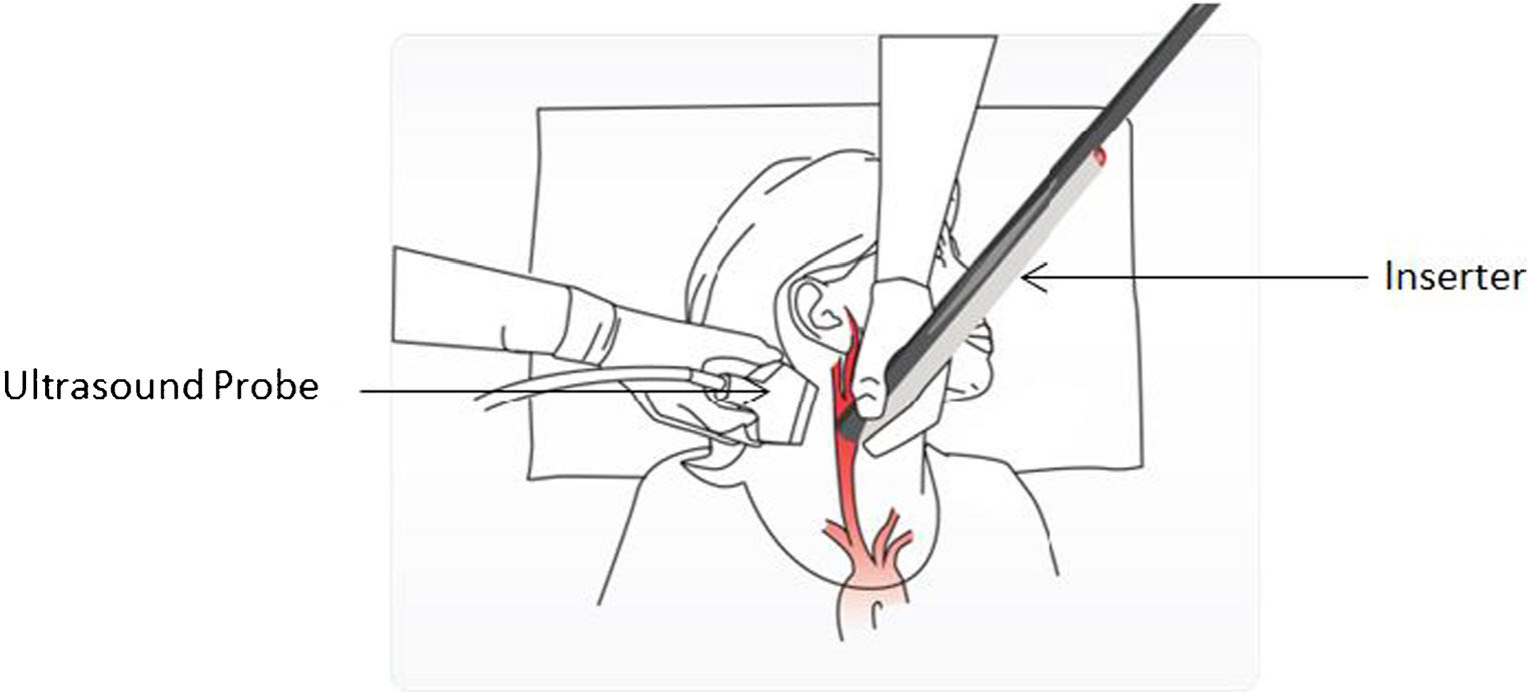
Implantation procedure positioning

The implant is deployed through the needle of an inserter under ultrasound imaging guidance (Fig. 4). The inserter is comprised of a Needle Unit and a Motor Unit (Fig. 2). The Needle Unit includes a siliconized insertion needle with a flag indicating the needle bevel orientation and a plunger. The Motor Unit includes motor, gear, hardware, and software. Once the Motor Unit and the Needle Unit are connected, upon pressing the operating button, the motor and gear automatically drive the plunger that pushes the implant out of the needle.

## Preclinical Studies

### In vitro Flow Simulator Testing

The implant’s ability to capture potential emboli was evaluated in a pulsatile flow simulator which mimics human CCA blood flow—an accepted method to evaluate the performance of filtration devices [29–30]. A pulsatile pump was used to circulate a blood simulation (37% glycerol in water) within a silicone tube at time-varying flow rates, representative of the human CCA diameter and flow [31]. Nylon balls (round, firm, symmetric balls of fixed size of 1.2 mm) and polyvinyl alcohol flakes (designed to mimic the consistency of blood clot texture and shape, at size ranges of 1.2–1.4 mm and 1.4–1.6 mm) were individually (*n* = 30) released into the flow, and capture performance of implants was tested. In the flow simulator, the implant captured 100% of nylon balls at fixed size of 1.2 mm. For polyvinyl alcohol particles in two size ranges of 1.2–1.4 mm and 1.4–1.6 mm, capture efficiency was 92% and 100%, respectively.

### In vitro Captured Thromboemboli Integrity

Sheep blood was placed in silicone tubes of 3.0 mm diameter. After 2 days, the tubes were cut and clots were extracted. Using the flow simulator described above, these clots (*n* = 100) at 3–7 mm in length were injected into the manifold and their integrity was observed during capture by the implant. All (100%) of the captured emboli were firmly adhered to the filtering portion of the implant, and no (0%) embolus breakdown was observed (embolus was progressively dissolved, and no distal pieces were observed in the manifold).

### In vivo Sheep Animal Model Testing

Sheep were used for in vivo testing of the Vine™ system because sheep have high rates of implant thrombogenicity including hypercoagulability, reduced clot lysis, and lack of response to aspirin [32–36]. The following parameters were assessed in acute and chronic studies: procedural success, implant safety, retrieval with pulling wire, surgical removal, fate of captured emboli, and inserter handling and performance characteristics. Safety parameters were assessed by ultrasound and X-ray at follow-up and by histopathology and scanning electron microscope (SEM) at termination.

### Procedural and Implant Safety Studies

Sheep (*n* = 30) were selected based on their CCA diameter (mm). For each sheep, one implant was deployed under ultrasound imaging guidance in each CCA, using the inserter. Enoxaparin was administrated on implantation day and for 2 more days post implantation together with clopidogrel. After implantation, CCA ultrasound examination was conducted at follow-ups at 4, 12, 13, 23, and 31 weeks, while the sheep were awake. At termination, ultrasound (before and after anesthesia) and X-ray examinations were performed. All implantation sites were harvested before the animals were euthanized, and the samples were sent for histopathology and SEM analysis.

**Fig. 5:**
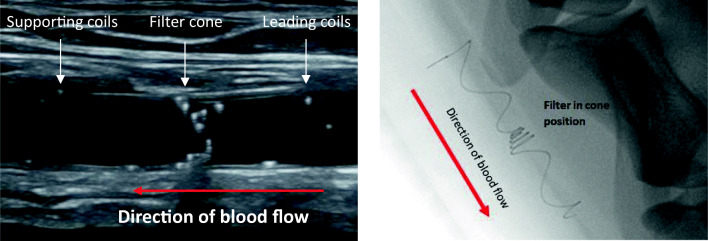
Ultrasound (left panel) and X-ray (right panel) images of implant

Overall, 30 of 30 animals (100%) survived and were in good health until sacrifice at the termination of the study. There were 64 implant deployments, of which 60 were successfully positioned. Procedural safety (lack of major bleeding, thrombus on implant, CCA damage or occlusion, and proper position of implant) was 64/64 (100%), and implantation success (proper implant position with a maximum of one implant retraction) was 60/60 (100%). In 4 cases of initial improper implant position, the implant was retrieved and successfully redeployed during the procedure. Representative images of the implant on ultrasound and X-ray are shown in Fig. 5.

**Fig. 6:**
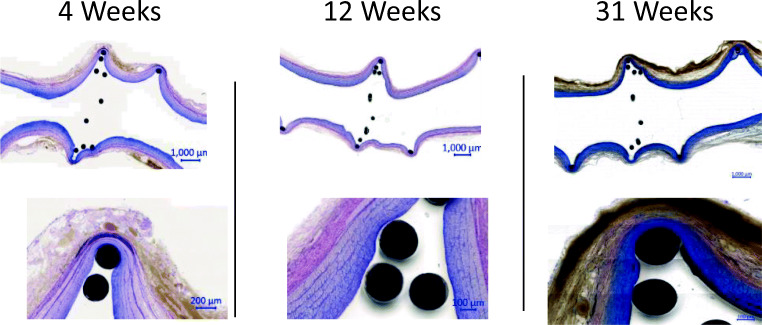
CCA segments, histopathology at termination (4, 12, and 31 weeks, H&E staining)

In histopathology analysis, no macroscopic abnormalities were observed at the implanted sites surrounding tissues and distant organs (lungs, liver, kidneys, spleen, heart, brain). The implants were correctly deployed and secured at the implantation sites in all specimens analyzed at 4 (10/10 implants), 12 (10/10), 13 (10/10), 23 (10/10), and 31 weeks (10/10). In two specimens (observed at 23 and 31 weeks), recent thrombi of sub-millimetric thickness were observed; occurrence of these thrombi was possibly related to pre-sacrifice/sacrifice procedure. There was no neo-intimal growth on the filtering portion wire. In some cases, there was partial neo-intimal growth on portions of the leading and supporting coils. Representative histopathology images are shown in Fig. 6. Black dots represent implant wire cross sections. Also note that the artery wall is not straight (zigzag) due to postmortem rigor mortis effect. There was minimal progression of neo-intimal growth over time (no difference between specimens at 1 month and up to 7 months).

**Fig. 7:**
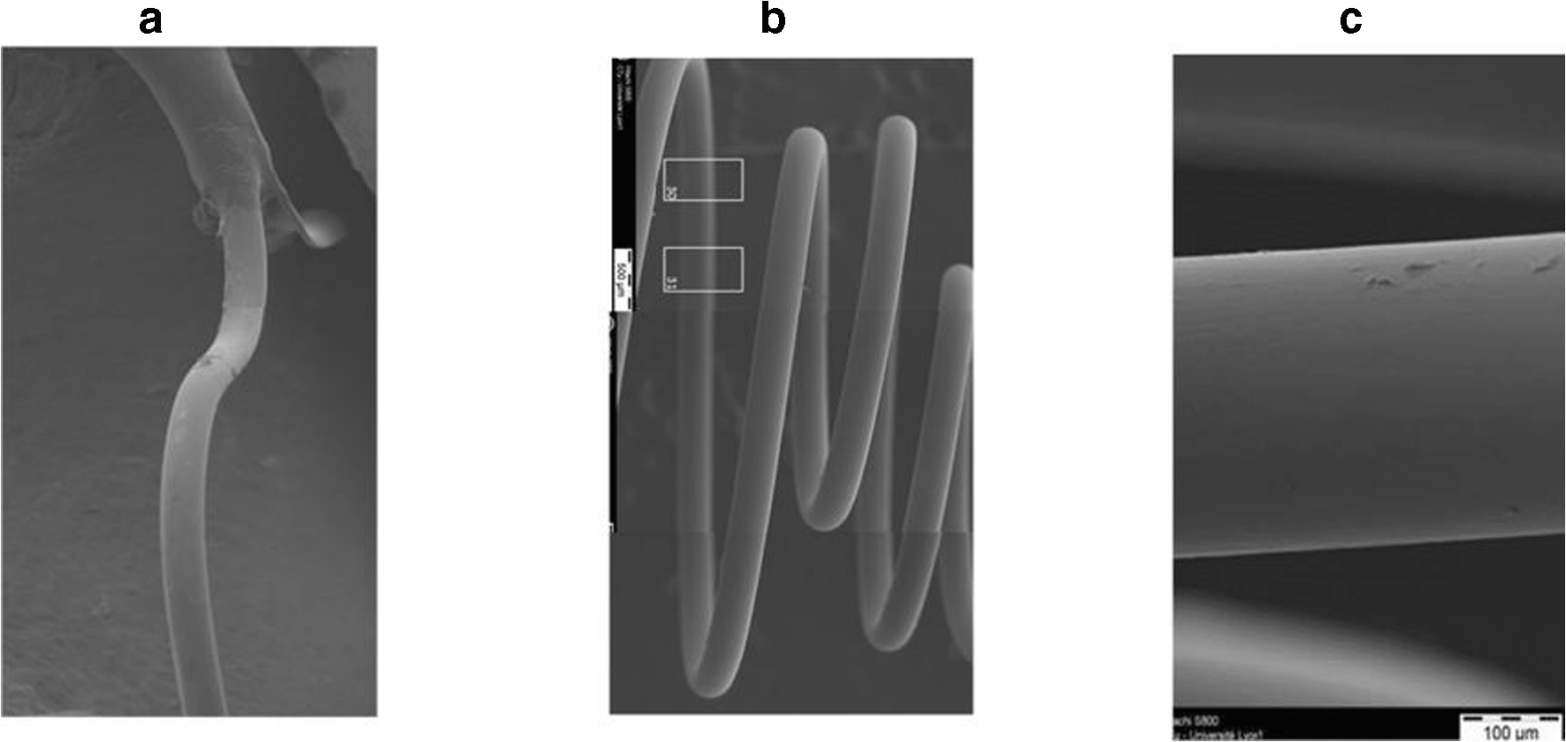
SEM images at termination (13 weeks): (**a**) Internal Anchor X40, (**b**) Filter X40, and (**c**) Filter Wire X200

The scanning electron microscope (SEM) analysis of all groups (*n* = 2, each) showed a correctly deployed and secured implant with only minimal signs of fibrin and blood cells deposited. No evidence of meaningful thrombus deposition was noted at the implant surface. The endothelial layer did not show abnormalities. Representative SEM pictures are shown in Fig. 7.

### Acute Retraction Study

In this study, implant retraction with the pulling wire (*n* = 12 implants) at 4 ± 0.5 h post implantation was achieved without difficulty using ultrasound guidance. This was done on the day of implantation and at day 12 (no vascular complications, no device integrity malfunctions). Gross pathology and histopathology findings at the CCA wall and surrounding tissue were minimal and observed in both retraction and control (puncturing only) sites.

### Late Extraction Study

Following implantation and cutting of the pulling wire, the implant can only be extracted by an endarterectomy-like surgical procedure. The procedure was done at 3 and 6 months post implantation (*n* = 14 implants and 4 implants, respectively). All implants were removed without vessel damage, and all sheep recovered from the procedure. In 1 animal, a systemic antibiotic was administered to treat a local wound infection.

### Fate of Emboli

The goal of this study was to assess the fate of captured thromboemboli in the sheep carotid artery. Implants were deployed bilaterally in 5 sheep (*n* = 10 implants). Three weeks after implantation, autologous thromboemboli (2 mm × 3–7 mm) were injected through a catheter at each CCA, and thromboemboli sizes were assessed by ultrasound at 1, 2, 3, 4, and 6 months following injection. Total regression of the captured embolus was seen in 4 (40%), 5 (50%), 7 (70%), 9 (90%), and 10 (100%) of cases, at each time point, respectively. There was no case of total obstruction to blood flow at any follow-up in 10 animals. In 3/10 arteries, thrombus on filter was very large (> 5 cm) at 1 month (2 cases) and 2 months (1 case). In these 3 arteries, temporary partial obstruction was observed, and implant shape was distorted. In all 3 arteries, normal blood flow resumed after thrombus regression. At final necropsy, no thrombi remnants were observed in any animal.

### Clinical Evaluation

The safety and effectiveness of the CCA filter system is being evaluated in three separate clinical studies, one completed, one ongoing, and the third planed:

The first in human study (CAPTURE 1, completed) was uncontrolled and included 25 patients with AF, with CHA_2_DS_2_-VASc score ≥ 2, unsuitable for OAC. Antithrombotic treatment consisted of aspirin + clopidogrel for 3 months, followed by aspirin lifelong.

The safety study, (CAPTURE 2, ongoing) is also uncontrolled and is planned to include 100 patients with AF and a previous history of ischemic stroke. Patients have a CHA_2_DS_2_-VASc score ≥ 4 and are receiving OAC (clinicaltrials.gov - NCT03892824). Antithrombotic treatment consists of OAC + clopidogrel + aspirin for 1 month, followed by OAC + clopidogrel for 5 months, followed by OAC as prior to implant.

A randomized controlled trial (INTERCEPT, planned to commence in 2021, will include 3500 patients, with CHA_2_DS_2_-VASc score ≥ 3, receiving OAC. It is a randomized superiority trial that will compare bilateral carotid filter implants to no carotid filter, on top of OAC in all patients, with a primary efficacy endpoint of ischemic stroke. There is no “dummy” procedure planned.

### First in Human Clinical Study (CAPTURE 1)

The first in human study, titled “Carotid Artery Implant for Trapping Upstream Emboli (CAPTURE 1) for Preventing Stroke in Atrial Fibrillation Patients,” was designed to assess the safety, feasibility, and tolerability of the system and implantation procedure in AF patients with CHA_2_DS_2_-VASc score ≥ 4 and deemed unsuitable for oral anticoagulants (OAC). The main exclusion criteria were CCA plaque in the “landing zone” and carotid stenosis > 30% in CCA, ICA, and ECA. The “landing zone” segment that needed to be free of plaque needed to be 4 cm in length (considering a 3.5–4 cm filter length). Study medications included heparin intra-procedure, clopidogrel + aspirin for 3 months, and aspirin lifelong. The study was designed as a multicenter, prospective, non-randomized, single arm, open-label study. Study outcomes were evaluated by an independent clinical events committee. A total of 48 patients underwent ultrasound screening: 23 patients (47%) were not enrolled due to either feasibility issues or withdrawal of consent. The remaining 25 patients had mean age of 71.3 years, 16 (74%) were male, and the mean CHA_2_DS_2_-VASc score was 4.4. Nearly half the patient cohort (48%) had a history of stroke, TIA, or thromboembolism.

**Fig. 8:**
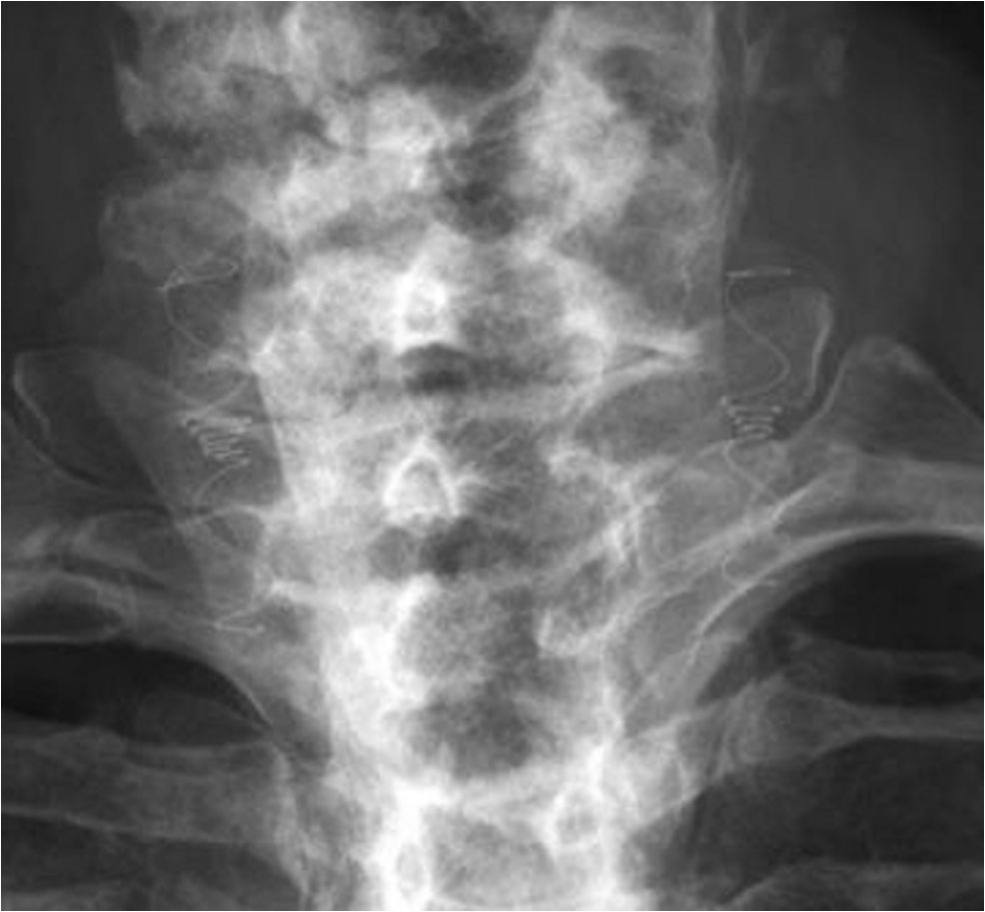
Vine™ filters in human CCAs

It was only possible to attempt filter implantation on both sides in 23/25 (92%) of patients due to poor imaging quality bilaterally (1 patient) and to previously unrecognized atheromatous plaque in the CCA on one side (1 patient). In the remaining 23 patients, the overall rate of initial successful deployment on the initial attempt was 47 out of 56 filters (84%). In the 9 patients with an initially unsuccessful implantation attempt, all devices (100%) were immediately retracted successfully using the pulling wire, and successful re-implantation was then performed. There were no major adverse events during the procedure. Of the 24 patients receiving an implant (23 bilaterally and one unilaterally), 24, 22, and 20 patients completed 3, 6, and 12 months follow-up, respectively (3 device-unrelated deaths and 1 patient lost to follow-up). No major device-related adverse events were seen during follow-up. Of the 47 implants (in 24 patients) that were properly positioned by the end of the procedure, all 47 (100%) remained properly positioned at all subsequent follow-ups. A typical X-ray image of bilateral correct filter positioning post implantation is shown in Fig. 8.

A puncture site skin hematoma occurred in several patients but was apparently related to soft tissue needle trauma, and no peri-CCA hematoma was observed in these cases by ultrasound imaging. In 3 of these cases, there was concomitant neck edema (confirmed by CT scanning), which possibly resulted from local reaction of soft tissue to blood oozing. All cases of hematoma/edema resolved by the 1-month follow-up visit without treatment. Three patients died of causes unrelated to the procedure or the device. One patient had two consecutive minor strokes, adjudicated as procedure/device unrelated, concomitant with unprovoked deep vein thrombosis (DVT). In both instances of stroke, MRI revealed several small infarcts in multiple brain territories including occipital lobes. Ultrasound imaging of that patient’s filters did not reveal thrombus on filter or any other abnormal finding.

Routine carotid ultrasound imaging was performed in all patients at 1 day, 1 week, and 1, 3, 6, and 12 months. There were 7 occurrences of asymptomatic thrombi on the filter in 4 patients, occurring between 1 and 9 months. No thrombus caused an interruption to blood flow as assessed by color Doppler ultrasound. Thrombi resolved completely within 0.5–5 months (with low molecular weight heparin in 3 and without in one). In one additional patient, a thrombus first observed at the final follow-up visit at 12 months appears to be regressing during extended follow-up.

## Discussion and Conclusions

Stroke prevention in atrial fibrillation remains an important medical need, despite the introduction of the highly effective non-warfarin oral anticoagulants. Absolute stroke risk remains unacceptably high in patients with AF patients with multiple risk factors (especially prior stroke), even when prescribed anticoagulation. This residual risk of cardio-embolism is partly due to inadequate dosing and to interruptions of anticoagulation for various reasons, including non-compliance and bleeding. Consequently, there is an unmet need for a sizable group of AF patients to improve stroke prevention. The permanent CCA filter is designed to provide protection additive to anticoagulation against anterior circulation stroke in such patients—who represent the vast majority of disabling strokes in patients with AF.

The relative success of the left atrial appendage occlusion device supports the concept of intravascular exclusion of embolism, but the left atrial occlusion device has several limitations compared with the vascular filter. It cannot exclude embolism coming from sources other than the left atrium, and implantation requires a major endovascular procedure with multi-disciplinary team and multiple imaging modalities to implant [37••, 38]. In addition, this device has not been tested as an adjunct to ongoing anticoagulation in high-risk AF patients [39].

In vitro and in vivo studies showed that the CCA filter captured thromboemboli which in turn completely resolved in time (weeks to months) and with no distal pieces observed in the manifold. This suggests that the risk of downstream embolization is low. The minimal neo-intimal growth is likely related to the relatively low radial force exerted by the implant on the carotid wall (~ 5 to 10% oversized relative to the CCA systolic diameter) as compared with carotid stents (30–100% oversized). The CCA filter is fixed in place by extra- and intra-luminal anchors; accordingly, migration is completely eliminated irrespective of the low radial force. The device can be readily implanted and retrieved (with pulling wire) and surgically extracted if necessary.

In a small study in patients with AF, device implantation and long-term follow-up was achieved with a high degree of safety. Ongoing and planned future studies will further define the safety and efficacy of this device for its intended use. The initial human study results indicate that deployment of a permanent coil filter in the human CCA is feasible and reasonably safe. The only potentially serious adverse effect was stroke, but the clinical picture suggested that this was not caused by the device. There were 4/25 patients in whom thrombus was detected on the implanted filters by routine ultrasound at various times during follow-up (7 cases), all occurring without stroke symptoms. In 6 cases, routine ultrasound imaging showed gradual but complete regression of thrombus over several months. In the 7th case, in which thrombus was detected late during follow-up (12 months), regression appears to be ongoing. Thrombus regressed while receiving sub-cutaneous heparin in 6 cases, and without in one case. It is not possible to know if thrombus detected on a filter is a “captured embolus” or an “in situ thrombus” formed on the filter. However, there are some clues that suggest that these were captured emboli. What was observed in sheep after injection of thrombus proximal to the filter was capture of these emboli by the filter, and these filter-associated thrombi had a string-like appearance. This is in contrast to the echogenic bulky mass appearance of in situ thrombus which formed on the filter in sheep immediately following implantation. The thrombi seen in patients had an appearance very similar to the string-like appearance of captured emboli in sheep, suggesting an embolic source. As observed in the sheep with captured emboli, we documented thrombus regression in all patients, presumably due to thrombolysis and the high shear force within the arterial circulation.

We observed a low procedural risk which is consistent with the evidence that inadvertent carotid artery puncture during jugular vein access rarely causes significant sequelae and that CCA punctures with needles smaller than 18G are benign [40–42]. The risk of inadvertent plaque rupture during carotid puncture was further mitigated by excluding atheromatous CCA segments using ultrasound screening. To reduce hematoma formation, we have introduced more meticulous application of local pressure at the puncture site for several minutes following implantation.

In summary, we have made significant progress in the development of a new device-based treatment for stroke prevention in AF. It is designed to be deployed easily with a high index of safety, and it is being developed as complementary to pharmacological treatment (rather than as an alternative) for higher risk AF patients. Extensive preclinical, as well as early human experience, suggests that the CCA filter could become a new device-based additive treatment for stroke prevention in AF. Ongoing and planned clinical trials are needed to further evaluate the efficacy and safety of this personalized preventive approach.

## Abbreviations

AF: Atrial fibrillation
CCA: Common carotid artery
OAC: Oral anticoagulants
NOAC: New oral anticoagulants
